# Temporal dynamics of abundant and rare microbial communities in fermented grains during Chinese light-aroma *Baijiu* fermentation

**DOI:** 10.3389/fmicb.2025.1640792

**Published:** 2025-10-16

**Authors:** Bin Lin, Wei Jiang, Jie Tang, Qun Li, Rui Li, Liping Zhu, Fan Zhang, Shengzhi Yang, Qiang Yang, Shenxi Chen

**Affiliations:** Hubei Provincial Key Lab for Quality and Safety of Traditional Chinese Medicine Health Food, Jing Brand Research Institute, Jing Brand Co., Ltd., Daye, China

**Keywords:** light aroma, abundant and rare taxa, microbial succession, microbial community assembly, molecular ecological network

## Abstract

The Chinese *Baijiu* fermentation process involves a complex multi-species microbial community, including both abundant and rare taxa. However, the rare and abundant microbes in fermented grains during *Baijiu* fermentation are poorly understood. In this study, we investigated the succession of abundant and rare microbial communities during Chinese light-aroma *Baijiu* (LAB) fermentation using the Illumina MiSeq and PacBio sequencing platforms. Our results showed that a total of 78 rare genera and 24 abundant genera were identified in the bacterial community. In comparison, 135 rare genera, 30 abundant genera, and two moderate genera were detected in the fungal community during the LAB fermentation. Abundant and rare microbial taxa showed significant differences in microbial diversity and taxonomic composition during two consecutive fermentation stages. At the later stage of grain fermentation, *Lactobacillus*, *Saccharomyces*, and *Condenascus* emerged as the dominant abundant genera. Unlike bacterial rare taxa (BRT), fungal dominant rare genera, including *Cladosporium*, *Saitozyma*, *Russula*, *Alternaria*, *Oidiodendron*, and *Chaetomium*, had an increasing trend throughout the fermentation process. Molecular ecological network analysis indicated that rare taxa, which accounted for more than 50% of keystone operational taxonomic units (OTUs), played a key role in maintaining the microbial community structure in fermented grain, and the bacterial network showed lower complexity than the fungal network at the late stage of grain fermentation. Moreover, the assembly of rare and abundant microbial communities was governed by stochastic processes. This study provides new insights into understanding the dynamic succession of abundant and rare taxa during LAB fermentation, thereby improving the quality and controllability of *Baijiu* production.

## Introduction

Chinese *Baijiu* is a traditional fermented liquor with a history spanning hundreds of years, representing a significant part of Chinese traditional food culture. It features rich flavor components and a multi-stage solid-state fermentation process (Jin et al., 2017; Sakandar et al., 2020; Yang et al., 2020). As the major type of *Baijiu*, light-aroma *Baijiu* (LAB) is popular among Chinese people due to its characteristic flavors, which include a soft, delicate, and refreshing taste (Sun et al., 2025). The entire brewing process of LAB involves *Jiuqu* making, grain saccharification, flavor fermentation, and distillation. After steaming and cooling, pretreated raw materials were mixed with mature *Jiuqu* powder, and then transferred to the fermentation tank for grain saccharification and flavor fermentation. Finally, the fermented grain was distilled to produce *Baijiu* after 14–30 days of fermentation, a duration determined by the type of starters (*Jiuqu*) and regional fermentation protocols (Tang et al., 2022).

The fermentation process of LAB largely relies on microbial metabolism, which can produce numerous flavor components that determine the quality of LAB (Shen et al., 2021). With the greatly improved accuracy and resolution of sequencing technology, the microbial composition in fermented grain can be thoroughly analyzed using a high-throughput sequencing method (Liu et al., 2024; Luo et al., 2024). A large amount of sequencing data reveals that the entire microbial community consists of a small number of abundant taxa and a large number of rare taxa, which could play a crucial role in maintaining a complex, multi-species microbial community (Wang Y. et al., 2020; Zhu et al., 2023). However, previous research on the microbiome involved in Chinese *Baijiu* brewing has generally focused on abundant microbial taxa due to their crucial roles in flavor formation during grain fermentation. Rare taxa are often overlooked, although they make up the majority of microbial diversity. Recently, rare microbiota in complex multi-species ecosystems have begun to receive widespread attention, and they have been reported to affect microbial structure and functions by altering microbial diversity (Chen et al., 2020; Ma et al., 2024; Dong et al., 2024). A comprehensive understanding of the changes in abundant and rare taxa is conducive to revealing the mechanism of microbial succession in fermented grains of Chinese *Baijiu*.

During natural microbial succession, abundant and rare microbial communities show distinct assembly processes in response to disturbances caused by both abiotic and biotic factors (Liang et al., 2020). Meanwhile, different microbial taxa can form a complex microbial network, in which the interactions between abundant and rare taxa may play a crucial role in regulating community function and structure stability (Hong et al., 2024). Thus, analyzing the potential microbial interactions coupled with the microbial assembly process could provide a clear understanding of microbial roles in maintaining the structure of ecosystem networks (Wang et al., 2018; Lin et al., 2024; Xiao et al., 2024; Yang et al., 2024). Previous studies have explored the mechanism behind the microbial assembly and network of rare and abundant taxa during the fermentation process of strong-flavor *Baijiu* and medium-temperature *Daqu* (Zhao et al., 2022; Song et al., 2024; Wen et al., 2024). However, a comprehensive analysis of the abundant and rare taxa succession involved in the microbial community during the *Baijiu* fermentation is still lacking. The LAB fermentation system offers considerable convenience in monitoring microbial changes throughout the entire fermentation process, and serves as an excellent experimental model for analyzing the microbial assembly during the multistage succession process (Shen et al., 2021).

In this study, we investigated the composition of fungal and bacterial communities throughout the entire fermentation process of LAB using next-generation sequencing technology, and further explored the changes of abundant and rare taxa within the fermentation ecosystem. We aimed to reveal that (1) the microbial succession pattern and assembly process of abundant and rare taxa during the LAB fermentation process; (2) the microbial interaction of abundant and rare taxa in the fermented grain LAB fermentation process. This research will provide essential information for improving the understanding of microbial mechanisms and the quality and yield of LAB.

## Materials and methods

### Sample collection

During the LAB production, the fermented grain samples were collected from a famous mechanized *Baijiu* factory in Daye, Hubei Province, China. Here, the entire brewing period of LAB lasts 15 days, including 1 day for grain saccharification and 14 days for flavor fermentation. The sorghum is incubated at 80 °C water for 18–20 h, and then sterilized twice for 30 min each. After cooling, it is mixed with *Jiuqu* and transferred to a cultivation chamber for grain saccharification. One day later, the saccharified grains are mixed with distilled grains and transferred to the fermentation tanks. The mixture was then subjected to anaerobic flavor fermentation for 14 days. The 500 g of fermented grain samples were randomly collected from the middle layer (approximately 0.75 m from the bottom of the tank) of each fermentation tank at fermentation days 0–5, 7, 9, 11, and 14. They were labeled as F0, F1, F2, F3, F4, F5, F7, F9, F11, and F14, respectively. Here, two independent production batches of samples from unified fermentation protocols were studied as two biological replicates. Three independent tanks were randomly selected from each production batch, and samples from three tanks at the same fermentation time for each batch were combined into a single sample for subsequent study. The samples were then stored at −80 °C for further microbial analysis.

### Total DNA extraction, polymerase chain reaction (PCR) amplification, 16S recombinant DNA (rDNA), and internal transcribed spacer (ITS) region amplicon sequencing

Total genomic DNA of fermented grain samples was extracted by using the Fungal/Bacterial Genomic DNA Extraction Kit (Solarbio Life Science, Beijing, China) according to the manufacturer’s instructions. In this study, for high-resolution taxonomic identification, the fungal community was profiled by sequencing a partial segment of the internal transcribed spacer (ITS) region (ITS2), whereas the bacterial community was characterized through sequencing of the full-length 16S ribosomal DNA (16S rDNA) region. The whole bacterial 16S rDNA region was amplified with barcoded primers 16S-F (5′-ACTCCTACGGGAGGCAGCA-3′) and 16S-R (5′-GNTACCTTGTTACGACTT′), and the fungal internal transcribed spacer 1 (ITS1) region was amplified with ITS1-F (5′-GGAAGTAAAAGTCGTAACAAGG-3′) and ITS1-R (5′-GCTGCGTTCTTCATCGATGC-3′). All PCR reactions were carried out in a 30 μL reaction system with 2 × PCR mixture reaction solution (Takara Bio, Beijing, China), 10 ng of template DNA, and 0.2 mM of each primer. The PCR conditions of 16S rDNA and ITS1 region amplification were performed as described in a previous study (Lin et al., 2022). Subsequently, PCR products were purified with the EasyPure QuickGel Extraction Kit (TransGen, Beijing, China). The purified DNA amplicons, each containing a different barcode, were pooled, and sequencing was performed using the Illumina MiSeq platform and the PacBio platform of Personal Biotechnology Co., Ltd. (Shanghai, China).

### Bioinformatics analysis

For the ITS region, raw data were first quality-filtered using Trimmomatic (version 0.33), and primer sequence was identified and removed by Cutadapt (version 1.9.1). High-quality sequences were obtained by splicing the double-ended reads using FLASH (version 1.2.11) and removing chimeras using UCHIME (version 8.1, Edgar et al., 2011). Subsequently, representative sequences of operational taxonomic units (OTUs) were obtained by clustering sequences at a similarity of 97%, and all sequences were filtered at a threshold of 0.005%. For the full-length sequencing of 16S rDNA, the circular consumption sequencing (CCS) sequence was obtained by correcting the original offline subreads using SMRT Link (version 8.0). Then, the CCS sequences of different samples were identified using barcode sequences, and chimeras were removed to obtain high-quality CCS sequences using lima software (version 1.7.0). Amplicon sequence variants (ASVs) are clustered and denoised by default with a threshold of 0.005% by the DADA2 method in QIIME2 (version 2020.6; Callahan et al., 2016). The representative sequences of ASVs or OTUs with the highest frequency were annotated using the SILVA database^1^ for bacterial species annotation and the UNITE database^2^ for fungal species annotation using an identity threshold of ≥97% (Edgar 2004; Quast et al., 2013). Raw sequencing data have been uploaded to the Short Read Archive database of the National Center for Biotechnology Information^3^ under the number BioProject PRJNA 1235431.

### Statistical analysis

Abundant and rare microbes in fermented grains samples were classified based on previous studies: (1) abundant taxa (AT) includes (a) always abundant taxa (AAT), which has a relative abundance of >1% in all samples, (b) conditionally abundant taxa (CAT), which has a relative abundance of >1% in some samples and >0.01% in all samples, and (c) conditionally rare and abundant taxa (CRAT), which has a relative abundance ranging from <0.01 to >1%; (2) rare taxa (RT) includes (a) always rare taxa (ART), which has a relative abundance <0.01% in all samples, and (b) conditionally rare taxa (CRT), which has a relative abundance <1% in all samples and <0.01% in some samples; (3) moderate taxa (MT) referring to OTUs or ASVs with a relative abundance between 0.01 and 1% in all samples (Xu et al., 2021; Zhao et al., 2022). The principal coordinates analysis (PCoA) was performed to evaluate the similarity in microbial communities between different samples using the “ape” package of R (version: 4.4.2) with ADNOSIS difference analysis. Molecular ecological network analysis was performed to study connections within abundant and rare microbial taxa using the ggClusterNet pipeline and visualized by R. The correlations with Spearman’s |*ρ*| > 0.8 and *p* < 0.05 were only shown in the network plot. The keystone OTUs in microbial networks were determined based on the topological parameters of nodes in networks, including within-module connectivity (Zi) and among-module connectivity (Pi) (Xu et al., 2021).

Assembly processes of abundant and rare communities were evaluated by using an ecological null model (permutations = 999) (Stegen et al., 2013); β-nearest taxon index (βNTI) was used to estimate the role of stochasticity and selection for community assembly using the “picante” package in R. According to the value of βNTI and Raup–Crick Bray–Curtis index (RC_bray_), stochastic or deterministic ecological processes were determined as the follows: (1) heterogeneous selection (βNTI > 2); (2) homogeneous selection (βNTI < −2); (3) dispersal limitation (|βNTI| < 2 and RC_bray_ > 0.95); (4) homogenizing dispersal (|βNTI| < 2 and RC_bray_ < −0.95); (5) undominated process (|βNTI| < 2 and |RC_bray_| < 0.95) (Shen et al., 2021).

## Results

### Microbial diversity of abundant and rare taxa in fermented grains during the LAB fermentation

**Figure 1:**
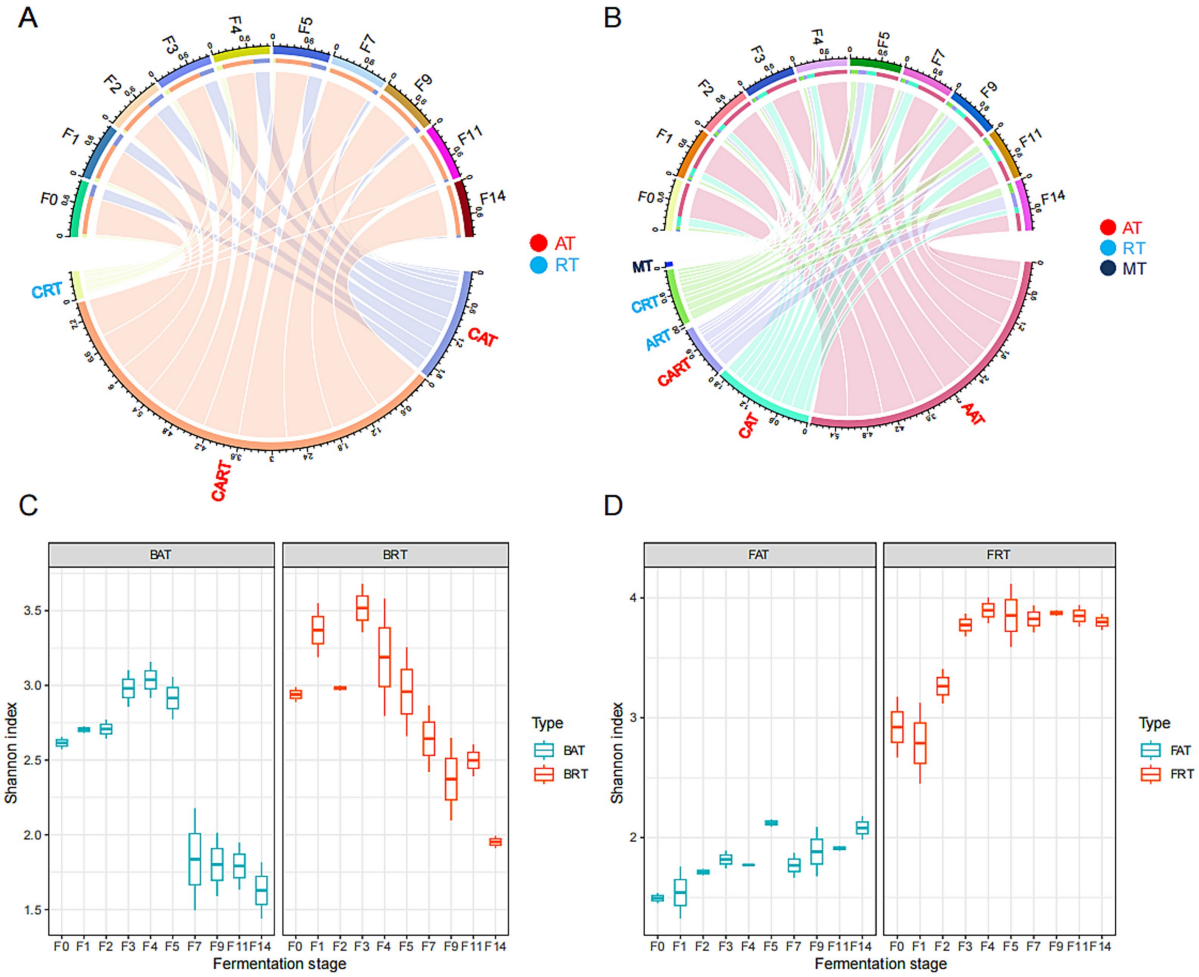
Microbial composition and diversity in fermented grains during the different fermentation times of the light-aroma *Baijiu* fermentation. The average relative abundance of rare and abundant taxa are shown in the chord diagram at the OTU or ASV level: **(A)** bacteria; **(B)** fungi. The Shannon index of abundant and rare microbial taxa along the fermentation process are shown in a boxplot: **(C)** bacteria; **(D)** fungi.

Rarefaction analysis indicated that the current sampling depth was sufficient for revealing the majority of fungal and bacterial diversity across all fermented grain samples (Supplementary Figure S1). A total of 115,933 CSS were obtained from 16S rDNA full-length sequencing after quality filtering and then assigned into 190 bacterial amplicon sequence variants (ASVs) at 97% sequence similarity. According to the definition mentioned in the method section, 190 bacterial ASVs were further categorized into 151 CRT, 7 CAT, and 32 CART. Among them, rare (CRT) and abundant taxa (CAT and CART) accounted for 0.74–17.8% and 82.1–99.2% of total bacterial relative abundance, respectively (Figure 1A). For the fungal community, a total of 1,070,782 high-quality sequence reads were obtained from ITS1 rDNA after quality filtering, then assigned into 507 fungal operational taxonomic units (OTUs) at 97% sequence similarity. These 507 OTUs were further categorized into 449 CRT, 26 CART, 14 ART, 12 CAT, 3 AAT, and 3 MT, based on their relative abundance. Among them, 463 rare OTUs, including CRT and ART, accounted for 2.7–23.46% of total fungal relative abundance, while 62 abundant OTUs, including AAT, CAT, and CART, accounted for 76.54–97.3% of the total relative abundance (Figure 1B).

The Shannon diversity index revealed that the alpha diversity of both abundant and rare microbial communities exhibited similar phase heterogeneity during LAB fermentation (Figures 1C,D). The diversity of both abundant and rare bacterial communities decreased along the fermentation process, and rare taxa had significantly higher diversity than abundant taxa during the whole-grain fermentation. However, the diversity of abundant and rare taxa in the fungal community increased significantly at the later stage of fermentation, and higher fungal diversity was found in the rare fungal community. Additionally, a higher Shannon index for rare bacterial and fungal communities indicated that rare taxa might be the major contributors to microbial diversity in fermented grains.

### Temporal dynamics of abundant and rare taxa during the LAB fermentation

**Figure 2:**
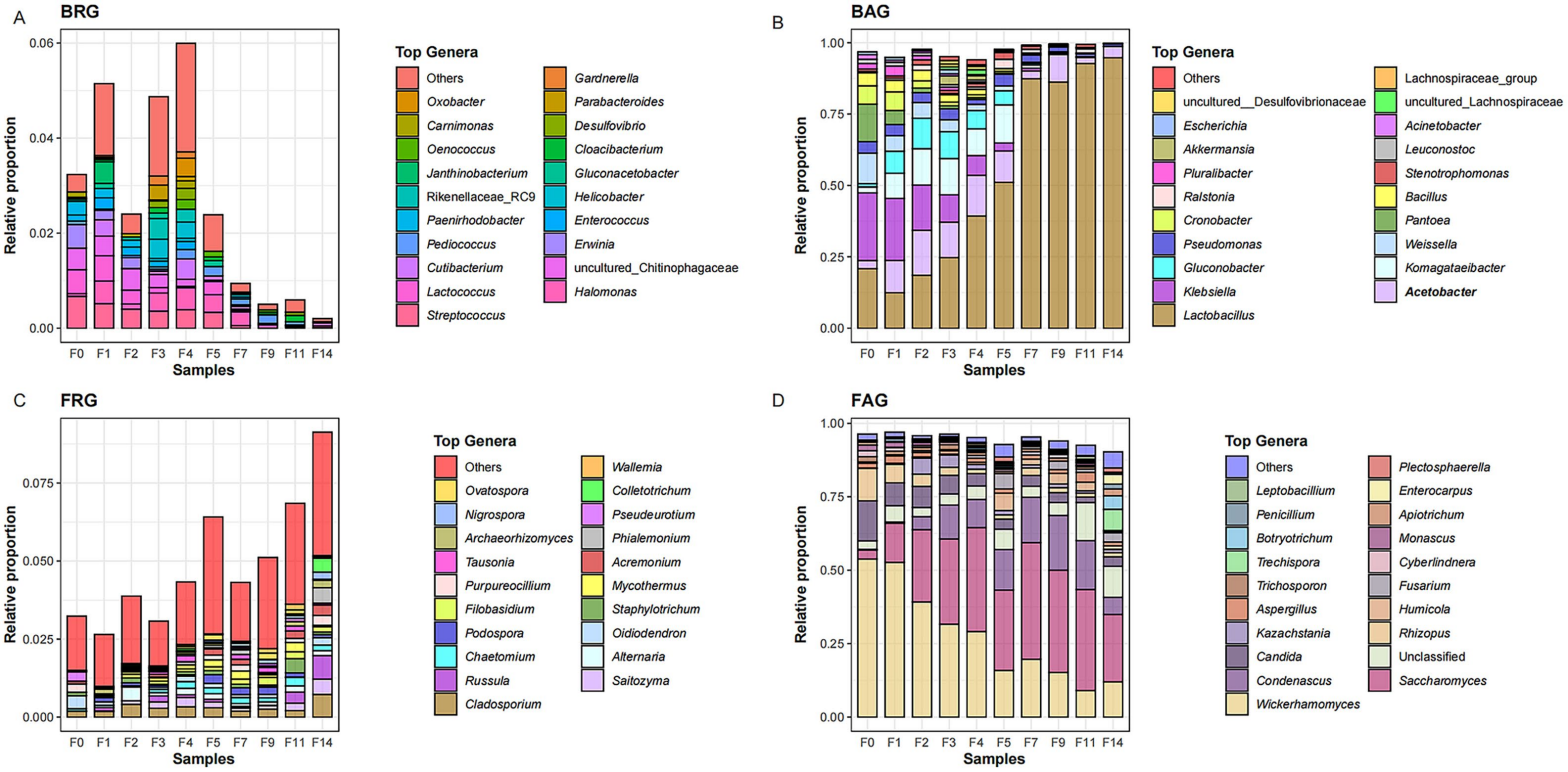
The composition of abundant and rare microbial taxa at different fermentation times of the light-aroma *Baijiu* fermentation: **(A)** bacterial rare genera (BRG); **(B)** bacterial abundant genera (BAG); **(C)** fungal rare genera (FRG); **(D)** fungal abundant genera (FAG).

To further understand the role of different microbial groups in microbial succession, we further analyzed the changes of the abundant and rare microbial genera during the fermentation of LAB. For the bacterial community, a total of 188 bacterial ASVs were categorized into 78 rare and 24 abundant genera through blasting against the SILVA database. During the LAB fermentation, as the major abundant genera, *Lactobacillus* (12.3–51.1%), *Acetobacter* (2.86–15.7%), *Klebsiella* (2.82–23.7%), *Komagataeibacter* (2.06–13.3%), *Gluconobacter* (1.18–10.8%), *Weissella* (1.66–10.7%), and *Pseudomonas* (1.70–4.14%) had high relative abundance of 1.7–51% at the fermentation days 0–5. After that, *Lactobacillus* accounted for 87.3–94.7% of total bacterial relative abundance as the absolute dominant genus, while the other six genera only accounted for 3.4–12.3% at the later stage of grain fermentation. *Streptococcus* (0–0.67%), *Lactococcus* (0–0.5%), *Halomonas* (0.03–0.47%), *Pediococcus* (0–0.19%), *Cutibacterium* (0–0.43%), and *Cloacibacterium* (0–0.13%) were identified as dominant rare genera, and their relative abundance decreased significantly at the later stage of LAB fermentation (Figures 2A,B).

For the fungal community, a total of 522 fungal OTUs were further categorized into 135 rare and 30 abundant genera through blasting against the UNITE database. At the initial fermentation stage, *Wickerhamomyces*, *Candida*, and *Rhizopus* were the dominant abundant genera, with relative abundance of 11.1–53.8%. As the grain fermentation proceeded, *Saccharomyces* (3–39%) and *Condenascus* (0.2–18.6%) became the dominant abundant genera. *Cladosporium* (0.18–0.72%), *Saitozyma* (0.18–0.72%), *Russula* (0.18–0.72%), *Alternaria* (0–0.44%), *Oidiodendron* (0–0.42%), and *Chaetomium* (0–0.27%) were identified as dominant genera among fungal rare taxa (FRT), and their relative abundance increased significantly at the end of grain fermentation (Figures 2C,D). Additionally, *Cutaneotrichosporon* and *Trichoderma*, as moderate taxa, accounted for 0.31–5.2% of the total relative abundance, and two moderate genera showed a slight fluctuation in relative abundance throughout the whole fermentation process.

**Figure 3:**
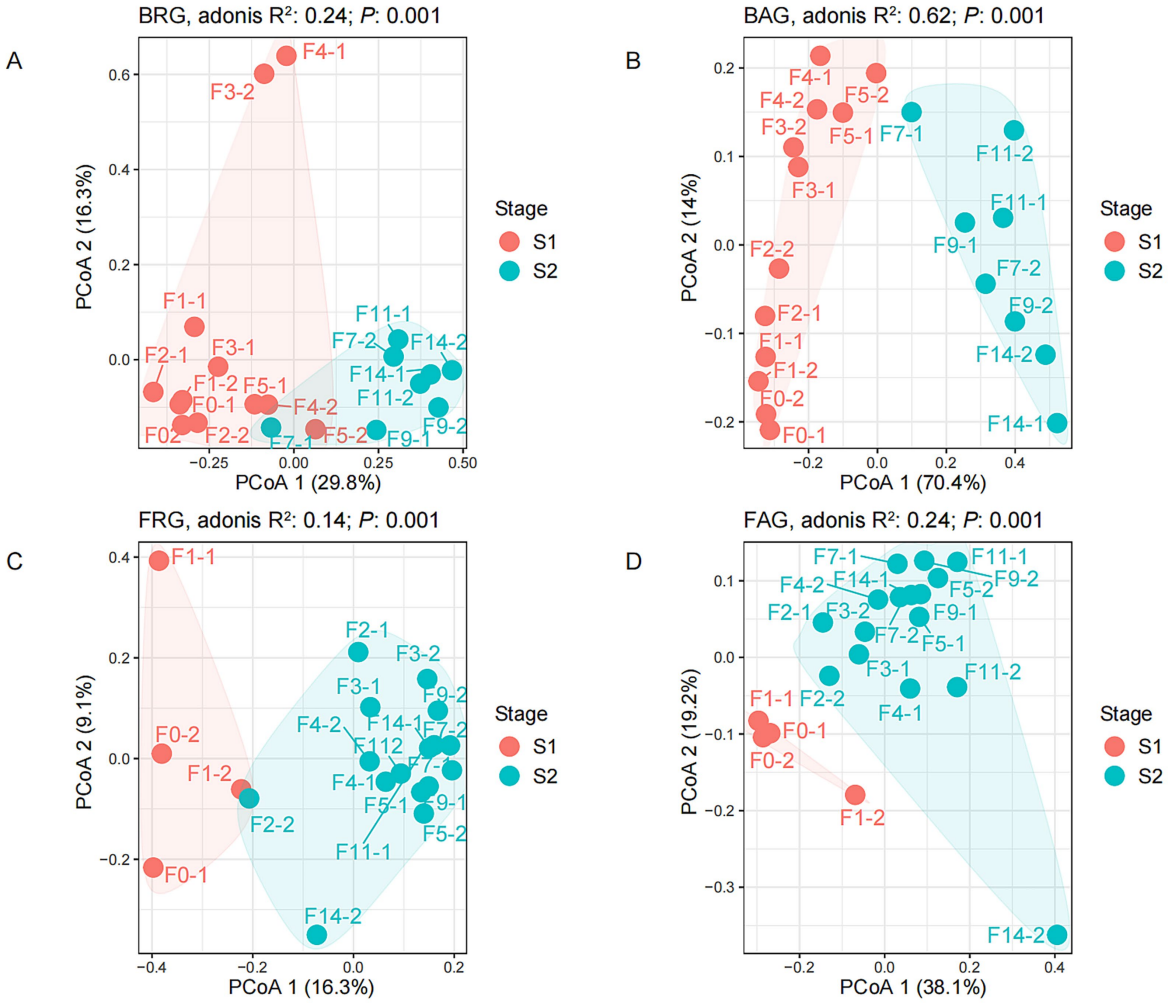
Principal coordinates analysis (PCoA) of rare and abundant microbial communities at different fermentation times. **(A)** Bacterial rare genera (BRG); **(B)** bacterial abundant genera (BAG); **(C)** fungal rare genera (FRG); **(D)** fungal abundant genera (FAG).

ADONIS analysis showed that the microbial composition structure changed significantly during the fermentation process (*p* = 0.001), and PCoA analysis also indicated that the microbial succession could be divided into two distinct fermentation stages (Figure 3). However, the fungal and bacterial communities changed significantly at the different fermentation time points. Abundant and rare fungal communities had significant differences in microbial composition after the second day of grain fermentation. In contrast, the significant changes in abundant and rare bacterial communities happened after the seventh day of grain fermentation. This result indicated that the fungal community (stage 1: 0–1 day; stage 2: 2–14 day) showed an earlier response to changes in the fermentation environment than the bacterial community (stage 1: 0–5 days; stage 2: 7–14 days).

### Molecular ecological network analysis of abundant and rare taxa in fermented grains during the LAB fermentation

**Figure 4:**
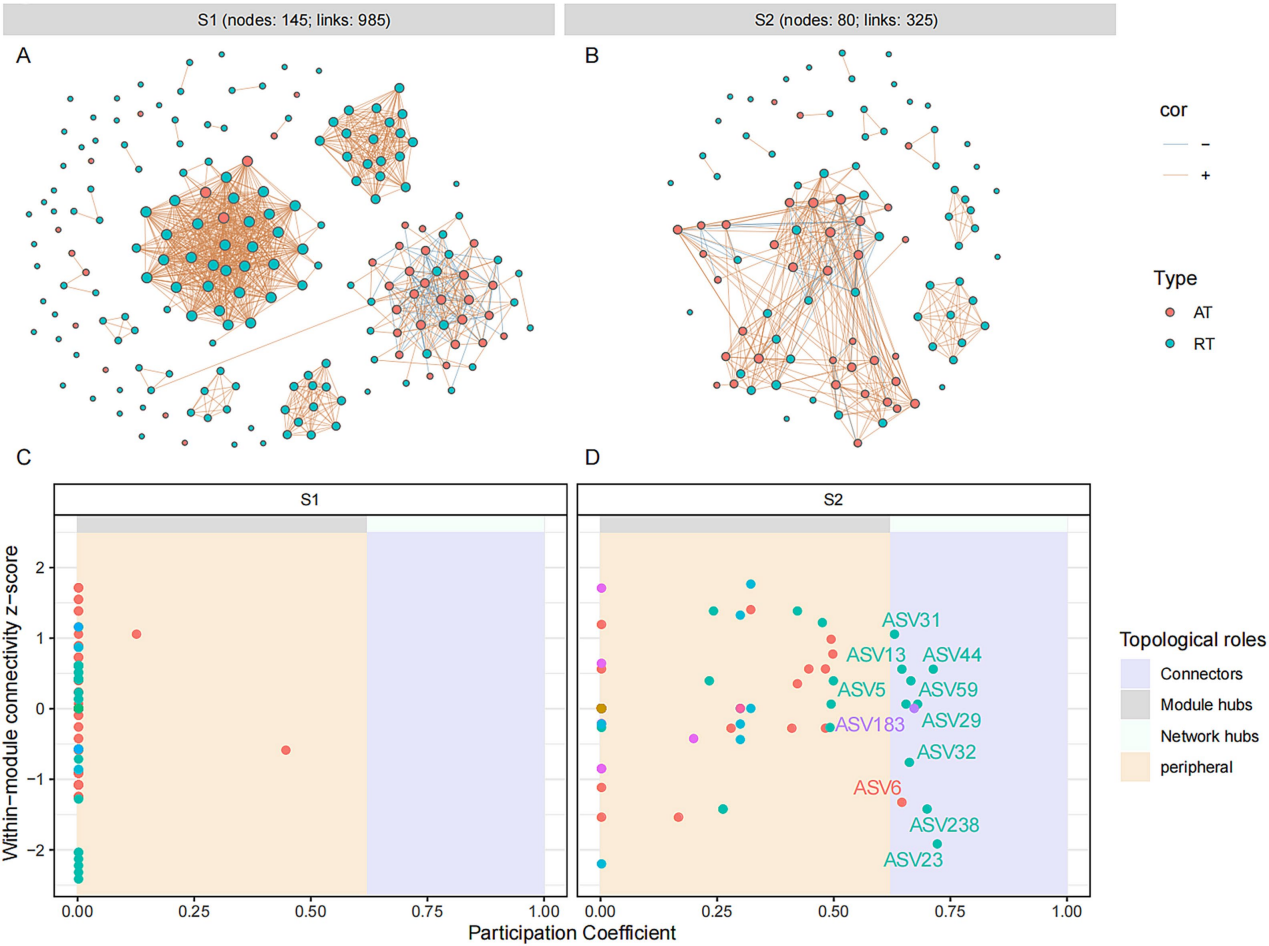
Microbial molecular ecological network and keystone taxa of bacterial community in different fermentation stages of the light-aroma *Baijiu* fermentation. **(A,C)**: stage 1; **(B,D)**: stage 2. Each node represents an ASV, and the size of the node is proportional to the degree of the ASV. The red and blue lines indicate positive and negative co-occurrence relationships with a significant correlation (*p* < 0.05), respectively. Keystone taxa are identified based on the topological properties of nodes in each network, with thresholds of intra-module connectivity (Zi) > 2.5 and/or inter-module connectivity (Pi) > 0.62.

**Figure 5:**
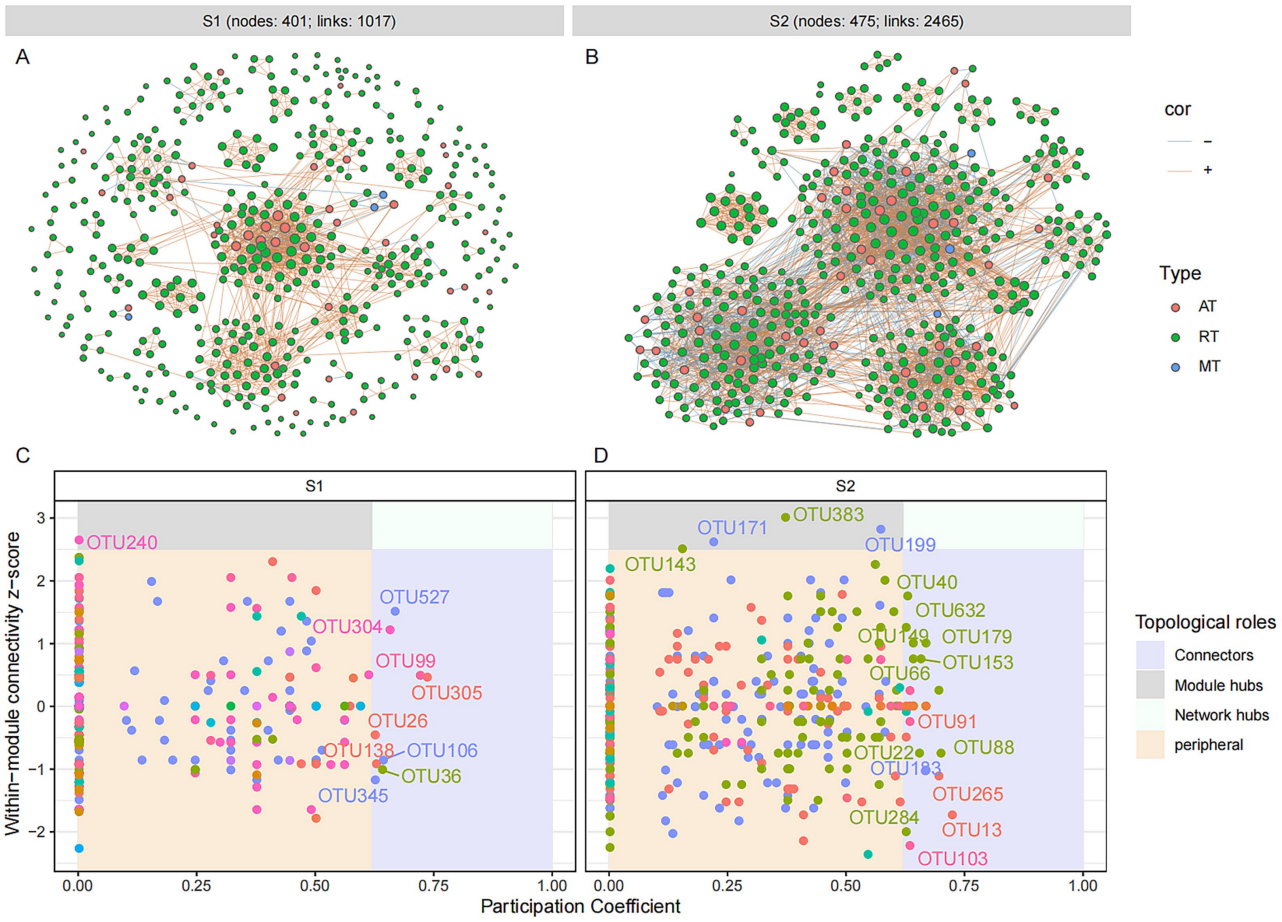
Microbial molecular ecological network and keystone taxa of fungal community in different fermentation stages of the light-aroma *Baijiu* fermentation. **(A,C)** stage 1; **(B,D)** stage 2. Each node represents an OTU, and the size of the node is proportional to the degree of the OTU. The red and blue lines indicate positive and negative co-occurrence relationships with a significant correlation (*p* < 0.05), respectively. Keystone taxa are identified based on the topological properties of nodes in each network, with thresholds of intra-module connectivity (Zi) > 2.5 and/or inter-module connectivity (Pi) > 0.62.

For uncovering the co-occurrence pattern of abundant and rare microbes in fermented grain samples at different fermentation stages, molecular ecological networks were constructed and visualized based on Spearman’s rank correlations (|*ρ*| > 0.8 and *p* < 0.05). A total of 985 and 325 pairs of correlations were identified from the bacterial network during the different fermentation stages (S1: 92% positive correlations; S2: 88% positive correlations), respectively (Figures 4A,B). A total of 1,017 and 2045 pairs of correlations were identified from the fungal network at the different fermentation stages (S1: 90% positive correlations; S2: 69% positive correlations), respectively (Figures 5A,B). The number of edges in the fungal network increased during the fermentation process, while those in the bacterial network decreased at a later stage of fermentation, indicating that the fungal group might play a crucial role in maintaining the stability of the microbial community at a later fermentation stage. The same variation was observed in the number of points in fungal and bacterial networks. For the bacterial molecular ecological network, rare OTUs accounted for 78.6 and 56.3% of total nodes (S1: 145; S2: 80) at different fermentation stages, respectively. Similarly, the fungal molecular ecological network contained a total of 401 fungal nodes in S1 and 405 fungal nodes in S2, among which 90.9 and 91% of fungal nodes were rare OTUs. According to the topological parameters (edges and nodes) of networks, the interactions of rare and abundant taxa had a remarkable change during different stages of grain fermentation.

The roles of microbial OTUs in microbial ecological networks were further evaluated by their within-module connectivity (Zi) and among-module connectivity (Pi) values (Figures 4C,D and 5C,D). ASVs or OTUs in module hubs and connectors are usually identified as keystone taxa that have a potential impact on modulating microbial structure and maintain community stability (Yuan et al., 2021). In the bacterial network, a total of 11 connectors were assigned to the keystone taxa at different fermentation stages (S1: 0; S2: 11), which were identified as *Lactobacillus*, *Pseudomonas*, *Pantoea*, *Halomonas*, and *Bacteroides* (Supplementary Table S1). Five connectors belonging to rare taxa were found among these bacterial connectors. In the fungal ecological network, nine connectors and one module hub were found in grain fermentation stage 1, and 25 connectors and four module hubs were detected in grain fermentation stage 2. The keystone taxa represented by connectors and hubs were belonged to 21 genera, among which 48% were identified as unclassified genera, and 92.3% of fungal keystone OTUs belonged to rare taxa. Moreover, higher-proportioned rare fungal keystone OTUs suggested that rare taxa had a key role in maintaining the stability of fungal network structure (Banerjee et al., 2018).

### Assembly processes of microbial rare and abundant communities during the brewing process

**Figure 6:**
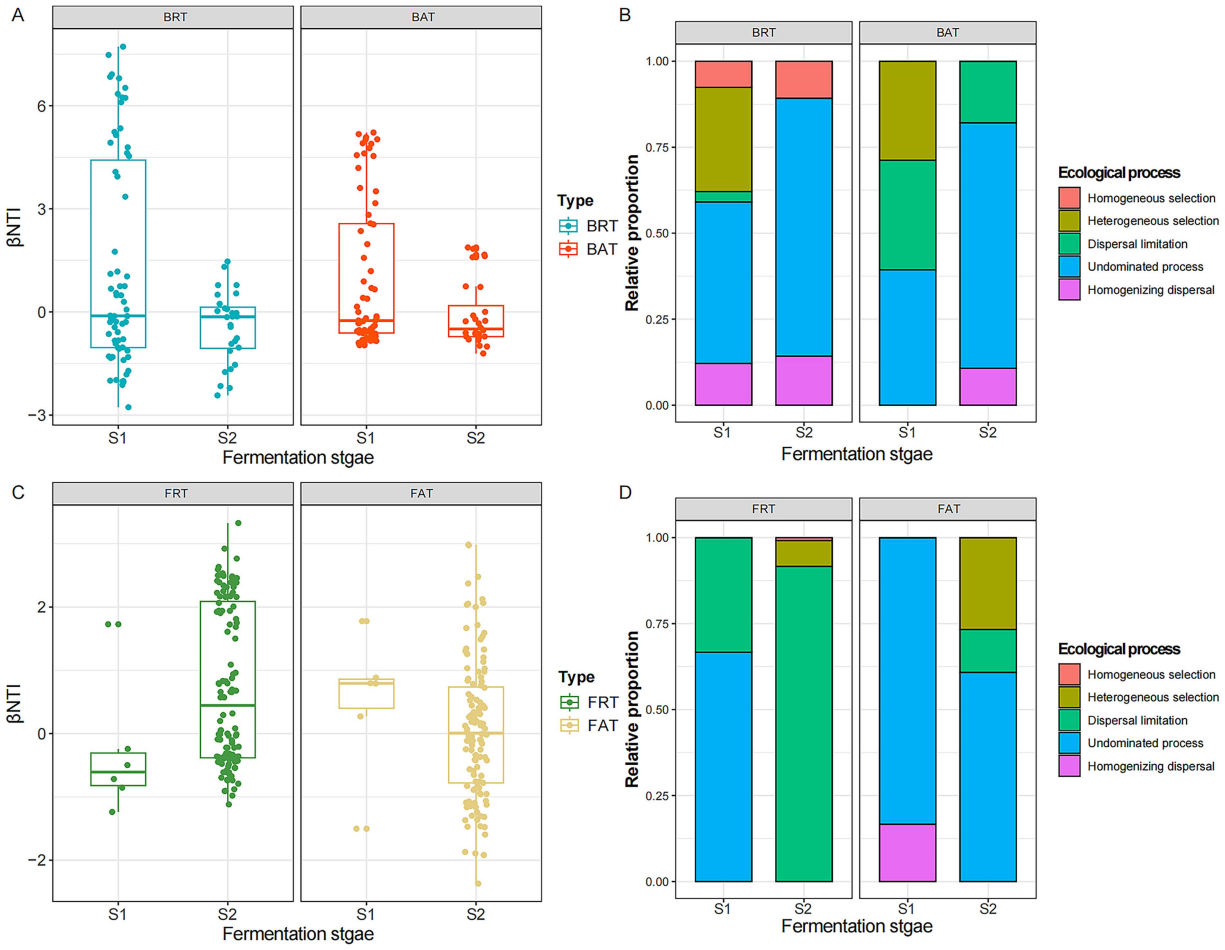
Ecological processes of abundant and rare microbial community assemblies during the different fermentation times. The βNTI value of rare and abundant bacterial **(A)** and fungal **(B)** communities during the different fermentation stages. The relative proportion of five ecological processes occurred in the rare and abundant bacterial **(C)** and fungal **(D)** communities. Fungal rare taxa (FRT); fungal abundant taxa (BAT); bacterial rare taxa (BRT); bacterial abundant taxa (BAT).

The β-mean nearest taxon index (βNTI) was used to assess the potential roles of deterministic and stochastic processes in the assembly of rare and abundant microbial communities at different stages of LAB fermentation (Figures 6A,C). In the rare bacterial community, the βNTI value ranged from 7.72 to −2.77, with 70.2% of βNTI values falling between −2 and 2. Similarly, the βNTI value of the abundant bacterial community for all possible pairwise combinations varied from 5.22 to −1.21, with 79.8% of βNTI values falling between −2 and 2. For the fungal community, the βNTI value ranged from 3.32 to −1.24 and 2.98 to −2.37 in the rare and abundant communities, with only 74.6 and 92.1% of the βNTI values being between −2 and 2. These results indicated that the assembly of rare and abundant fungal and bacterial communities depended on stochastic effects and was not significantly affected by the abiotic factors in the external environment.

We further evaluated the relative contributions of major ecological processes governing the assembly of abundant and rare microbial communities at different fermentation stages (Figures 6B,D). According to the βNTI and RC_bray_ values, five different ecological processes (heterogeneous selection, homogeneous selection, dispersal limitation, homogenizing dispersal, and undominated process) were identified in the assembly of abundant and rare communities, and they had significantly different effects in shaping the composition of microbial communities. The results further showed that ecological process belonging to stochastic selection dominated the assembly of bacterial communities at different fermentation stages, with undominated process being the main driver of changes in bacterial community composition (rare: 47% in S1 and 75% in S2; abundant: 39% in S1 and 71% in S2), and heterogeneous selection also had a crucial role in shaping rare (30%) and abundant (29%) bacterial community structure in S1. Similarly, for fungal taxa, the assembly process of the rare community was dominated by the undominated process (83% in S1 and 61% in S2), and heterogeneous selection made a contribution much to the succession of the rare community in S2. In contrast to the dominance of undominated processes (66.7%) observed in S1, dispersal limitation emerged as the predominant driver for the assembly of the fungal abundant community in S2, accounting for 92% of the ecological processes, respectively.

## Discussion

Understanding the changes in rare and abundant taxa is essential for a comprehensive understanding of microbial succession in complex multi-species ecosystems (Zhu et al., 2023). Although the composition and functions of abundant and rare microbial taxa have been studied in recent years, little is known about their microbial diversity and assembly mechanism in fermented grains of Chinese *Baijiu*, especially LAB. Thus, disentangling the microbial variation and ecological roles of rare and abundant taxa is crucial for regulating microbial processes during the LAB fermentation.

Our study elucidated the characteristics of microbial succession in fermented grain during the LAB fermentation based on the composition of rare and abundant taxa. A previous study indicated that the fermentation processes of Chinese LAB underwent two stages based on microbial dynamics (Lin et al., 2022). Similarly, in this study, the abundant and rare microbial composition showed temporal heterogeneity in fermented grain across two consecutive stages of LAB fermentation: during the initial fermentation days, residual oxygen in the fermentation tank supported the short-term growth of aerobic abundant and rare taxa. As oxygen was depleted, anaerobic microorganisms—such as yeasts and lactic acid bacteria—began to proliferate rapidly. Notably, fungi may be more sensitive to environmental fluctuations in fermented grains. Thus, both abundant and rare fungal taxa showed distinct microbial succession patterns compared to their bacterial counterparts, and responded more quickly during the LAB fermentation (Lin et al., 2022). In complex multi-species ecosystems (such as soil and water), microbial rare taxa with less abundance make up the majority of species composition, and perform the key function to maintain ecosystem stability (Jia et al., 2018; Yuan et al., 2022). This phenomenon was also observed in microbial succession in fermented grain of LAB, in which rare OTUs accounted for 88.09% of the total number of microbial OTUs. It indicated that rare taxa contributed to microbial diversity and may play an important role in microbial networks during grain fermentation. Compared to rare bacterial taxa with decreasing relative abundance, fungal rare genera occupied a higher proportion at a later stage of grain fermentation, and they might contribute more to the microbial community than bacterial rare taxa during the fermentation process (Song et al., 2024). In addition, the increasing proportion of rare taxa may be attributed to the fast adaptation of special species to changes in abiotic factors in fermented grains. Rare taxa derived from *jiuqu* (stater), raw material, and workshop environment are more likely to be screened for *Baijiu* brewing through microbial antagonism during the *Baijiu* fermentation process, and these microbial taxa could undergo fast adaptation to the changes in the fermented container after the beginning of LAB fermentation (Zhao et al., 2022). Thus, the establishment of rare microbial consortia in fermented grains through specific environmental filtering might explain the increasing diversity of the rare fungal taxa at the later stage of grain fermentation.

Here, abundant taxa and rare taxa might have distinct roles and functions in the microbial community of fermented grain of LAB. *Wickerhamomyces*, *Saccharomyces*, and *Lactobacillus* were identified as the dominant abundant genera during the LAB fermentation, and they could hydrolyze macromolecules to generate substrates for the production of the key flavor components such as alcohols, acids, and esters (Wang et al., 2024). Unlike abundant taxa, rare taxa might play a crucial role in regulating the microbial community by changing the microbial interaction as keystone taxa, rather than flavor production (Song et al., 2024), which was supported by the high proportion of rare taxa in the microbial networks. Thus, as the fermentation environment stabilized, the number of rare OTU keystones in microbial networks increased, maintaining the stability of microbial networks at the later fermentation stage (Stage 2). In addition, rare taxa can also be regarded as a vast repository of microbial diversity in natural ecosystem (Gagic et al., 2015). Similarly, certain rare taxa possess the potential to become the abundant taxa in adapting to the high-acid environment formed in LAB fermentation, particularly *Lactobacillus* species. *Lactobacillus pontis* and *L. helveticus*, initially rare in the early stage of fermentation, emerged as the abundant taxa in the later stage of grain fermentation. These lactic acid bacteria can enhance the production of organic acids, ethanol, and flavor esters, thereby significantly contributing to the aromatic production of fermented foods (Wang X. Y. et al., 2020; Gao et al., 2024). Therefore, these rare taxa could serve as potential candidates for screening functional microbial strains applied in *Baijiu* production, especially for flavor enhancement through targeted microbial management tools.

Compared to the single-species fermentation system, microbial interactions played a crucial role in maintaining the stability of the microbial community in a multi-species fermentation system (Yao et al., 2022). The current study indicated that positive associations between microbial nodes dominated the microbial ecological network during LAB fermentation, and this phenomenon was also observed in the fermentation process of Chinese strong-aroma *Baijiu* (Zhao et al., 2022). The majority of microorganisms involved in LAB fermentation co-operate with each other, and their complex interactions can maintain the whole community structure in the fermented grain ecosystem and improve flavor production. As the dominant abundant taxa, *Wickerhamomyces* and *Saccharomyces* were reported to work with other microbial taxa to improve the production of the characteristic flavor substances (alcohols, acids, and esters) during the process of food fermentation (Gao et al., 2022; Zhang et al., 2024). In addition, the analysis of the topological parameters of the microbial ecological network (Supplementary Table S2) revealed that the fungal network, characterized by lower clustering coefficients and degree centralizations, exhibited closer associations between abundant and rare taxa compared to the bacterial network. These results suggested that the fungal network had higher complexity during the entire grain fermentation process coupled with the greater number of nodes and connections within the fungal network. A previous study reported that higher network complexity could lead to greater instability in microbial ecological networks (Wen et al., 2024). Thus, the fungal network in fermented grains, with its relatively weaker stability, may be more vulnerable to external environmental changes, which explains why the fungal community responded earlier during LAB fermentation.

Disentangling the contributions of ecological processes to microbial community assembly is a key issue in exploring microbial succession. In the natural ecosystem, stochastic and deterministic processes usually have distinct influences on the assembly process of abundant and rare microbial communities (Hou et al., 2022). However, interestingly, our results showed that the stochastic assembly largely controls the assembly process of rare and abundant microbial communities during the grain fermentation of LAB, especially the undominated process. An undominated process refers to multiple ecological processes, including diversification, drift, weak selection, and weak dispersal (Zhou and Ning, 2017). The dominance of the undominant process might be attributed to microbial domestication for special micro-ecology in fermented grain (Somerville et al., 2024). After a long period of brewing practices in the LAB distillery, the majority of the microbes involved in grain fermentation have adapted to the high acidity, low oxygen, and hypoxic environment in fermentation containers, especially fungi (Tong et al., 2024). Rare and abundant fungal communities responded rapidly to external disturbances just in the first 2 days of LAB fermentation, and special fungal taxa could have a rapid reproduction in fermented grain. A previous study also confirmed that the major filamentous fungi and ethanol-producing yeasts had high adaptability to fermentation environment, resulting in governing stochastic assembly in the fungal community succession of LAB fermentation (Shen et al., 2021). Therefore, the limited selective pressure on both rare and abundant microbial taxa caused by environmental disturbances during grain fermentation led to a predominantly stochastic community assembly process during the short-term fermentation of LAB.

## Conclusion

In conclusion, this study revealed the composition and changes of abundant and rare microbial taxa during the solid-state fermentation process of Chinese LAB by the amplicon sequencing technology. During the LAB fermentation process, abundant and rare microbial taxa showed significant differences in microbial diversity and taxonomic composition. Rare taxa were pivotal in maintaining microbial diversity and structure in fermented grain. Molecular ecological network analysis revealed that the fungal network, mainly composed of rare OTUs, exhibited significantly greater stability compared to bacterial counterparts during the later stages of grain fermentation, suggesting enhanced ecological resilience. Moreover, a stochastic process was identified as the dominant determinant governing the assembly mechanisms of the rare and abundant microbial communities. This work would provide valuable insights for microbial management policies based on rare and abundant microbial taxa, and improve the flavor quality of Chinese LAB.

## Data Availability

The datasets presented in this study can be found in online repositories. The names of the repository/repositories and accession number(s) can be found at: https://www.ncbi.nlm.nih.gov/, PRJNA1235431.

## Footnotes

^1^ http://www.arb-silva.de/

^2^ https://unite.ut.ee/

^3^ http://www.ncbi.nlm.nih.gov

## Supplementary material

SUPPLEMENTARY FIGURE S1 Rarefaction analysis of microbial communities in different fermented grain samples of light-aroma *Baijiu*: **(A)** bacteria; **(B)** fungi.
