# Supplementary figures and images for: Temporal dynamics of abundant and rare microbial communities in fermented grains during Chinese light-aroma Baijiu fermentation
Source: Front Microbiol. 2025 Oct 16;16:1640792. doi: 10.3389/fmicb.2025.1640792 (PMC12571738; doi:10.3389/fmicb.2025.1640792)

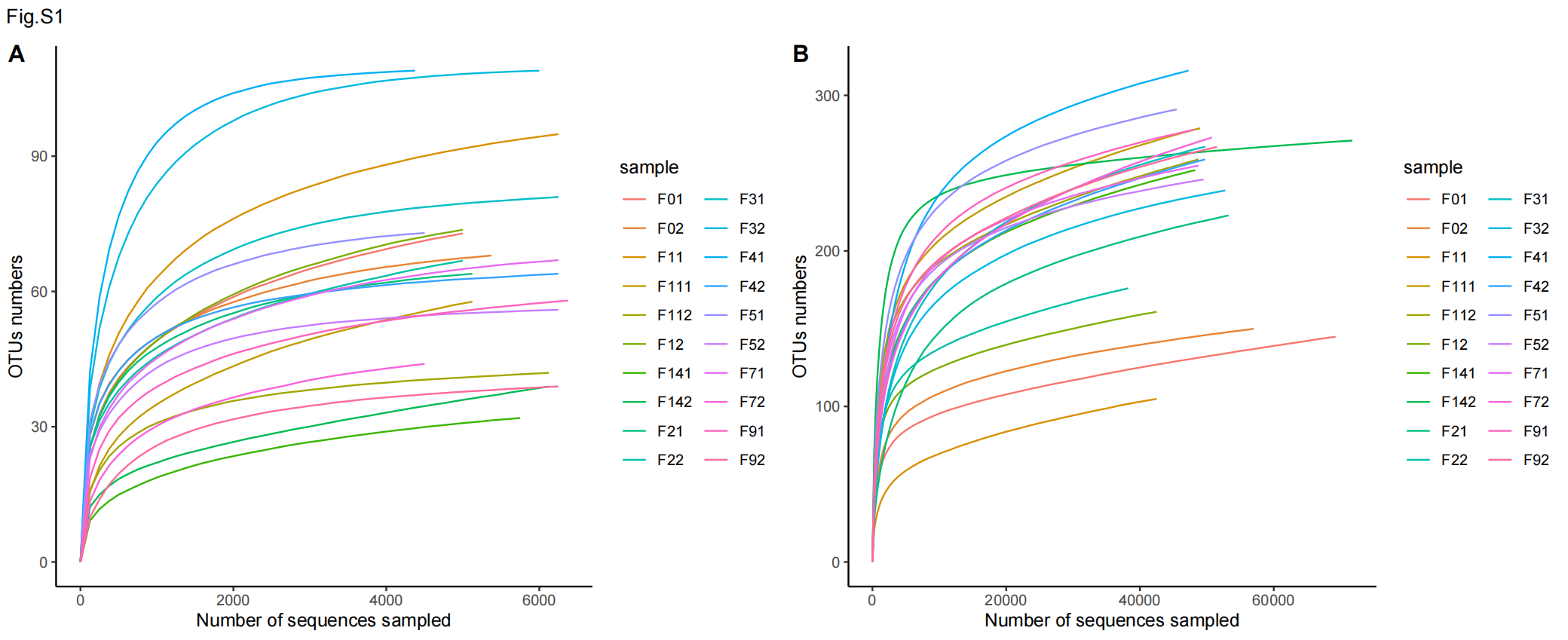

Supplement: SUPPLEMENTARY FIGURE S1 — Rarefaction analysis of microbial communities in different fermented grain samples of light-aroma Baijiu: (A) bacteria; (B) fungi. [file Image_1.TIF]
